# Intracerebral Hemorrhage-Induced Cognitive Impairment in Rats Is Associated With Brain Atrophy, Hypometabolism, and Network Dysconnectivity

**DOI:** 10.3389/fnins.2022.882996

**Published:** 2022-06-30

**Authors:** Laurent Puy, Clémence Leboullenger, Florent Auger, Régis Bordet, Charlotte Cordonnier, Vincent Bérézowski

**Affiliations:** ^1^Univ. Lille, Inserm, CHU Lille, UMR-S1172 – LilNCog - Lille Neuroscience and Cognition, Lille, France; ^2^Univ. Lille, CNRS, Inserm, CHU Lille, Institut Pasteur de Lille, US 41 - UMS 2014 - PLBS, Lille, France; ^3^UArtois, Lens, France

**Keywords:** stroke, cognitive impairment, MRI, PET, connectivity, intracerebral hemorrhage

## Abstract

The mechanisms underlying intracerebral hemorrhage (ICH)-related cognitive impairment (CI) remain unclear. Long-term structural and functional changes were investigated in the brains of healthy male and female Wistar rats after experimental ICH. Following double injection of autologous blood, rats underwent short-term (onset, 3 and 7 days) and long-term (3 and 6 months) radiological assessment and behavioral tests exploring spontaneous locomotion, anxiety-like behavior and working memory, spatial recognition memory and visual recognition memory. Volumetric and metabolic changes in brain areas were examined by 7Tesla-MRI and [18F] FDG-PET, respectively. Brain connectomic disorders and maladaptive processes were seeked through brain metabolic connectivity analysis and atrophy-related network analysis. From an initial hematoma mean volume of 23.35 ± 9.50 mm^3^, we found early spontaneous locomotor recovery and significant spontaneous blood resorption (≈ 40% of the initial lesion) from days 0 to 7. After 3 and 6 months, ICH rats exhibited CI in several domains as compared to the sham group (working memory: 58.1 ± 1.2 vs. 70.7 ± 1.2%, *p* < 0.001; spatial recognition memory: 48.7 ± 1.9 vs. 64 ± 1.8%, *p* < 0.001 and visual recognition memory: 0.14 ± 0.05 vs. 0.33 ± 0.04, *p* = 0.013, in female only). Rats that experienced ICH had remote and concomitant cerebral atrophy and hypometabolism of ipsilateral striatum, thalamus, limbic system and cortical areas (temporal and parietal lobes). Interestingly, both structural and metabolic deterioration was found in the limbic system connected to the affected site, but remotely from the initial insult. On the other hand, increased activity and functional connectivity occurred in the contralateral hemisphere. These connectomics results showed that both maladaptative and compensation processes coexist in the rat brain following ICH, even at young age and in a disease-free setting. These radiological findings deepen our understanding of ICH-related CI and may serve as biomarkers in the view of future therapeutic intervention.

## Introduction

Spontaneous intracerebral hemorrhage (ICH) is among the most severe forms of stroke, accounting for almost half of stroke-related morbidity and mortality (Poon et al., 2014). ICH survivors are also exposed to cognitive impairment (CI), one third of them will develop new onset dementia in the ensuing years (Moulin et al., 2016). Recent data offered a better characterization of CI that affected several cognitive domains, such as language, memory, executive functions, processing speed and visuo-spatial abilities (Donnellan and Werring, 2020). However, the underlying mechanisms of ICH-related CI has yet to be established, leaving patient without specific treatment. In particular, the long-term cerebral structural and functional correlates of ICH-related CI need to be further addressed. In clinical setting, disentangling the sole effect of bleeding on cognitive function from the coexisting presence neurodegenerative and/or small vessel diseases (such as cerebral amyloid angiopathy) is impossible (Donnellan and Werring, 2020; Pasi et al., 2021). Experimental models offer a unique opportunity to investigate the changes occurring in the brain of animals with ICH-related CI that can be monitored in the view of future pharmacomodulation. In the current study, we investigated the long-term hippocampal and non-hippocampal cognitive effect of ICH in healthy male and female rats, devoided of underlying cerebrovascular disease. To deepen our understanding of ICH-related CI, we concurrently studied the long-term structural (7T-MRI and automated brain atrophy segmentation) and functional ([18F] FDG PET) changes that occur in the rat brain. We performed brain metabolic connectivity analysis to further investigate the brain connectomics disorders and adaptative process that occur in the rat after ICH.

## Materials and Methods

### Ethical Aspects

All experiments were approved by the national Ethical Committee in Animal Experimentation (CEEA, Comiteì d’Ethique en Experimentation Animale), from the French Ministry for Education and Research (agreement number: APAFIS#14066-2018031312529642v3) and were performed in strict compliance with the European Union Directive 2010/63/EU. Experiments were reported in accordance with the ARRIVE guidelines for reporting experiments involving animals.

### Animals and Study Design

**FIGURE 1:**
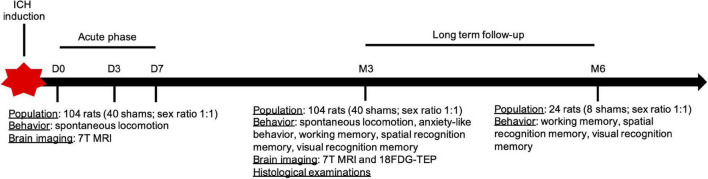
Design of the study. ICH, intracerebral hemorrhage.

We used 12-week-old Wistar rats and included both males (280–350 g) and females (220–300 g). We prospectively studied 114 male and female rats: 74 in the ICH group (sex ratio 1:1) and 40 in the sham group (sex ratio 1:1). After surgery, each rat underwent short-term (days 0, 3, and 7) and long-term (3 and 6 months) behavioral and radiological assessments. Details of the study design are reported in Figure 1.

### Intracerebral Hemorrhage Induction

Animals were anesthetized with isoflurane (1.5–2%) through spontaneous respiration and core temperature was maintained at 36–37°C throughout all surgical procedures. A stereotaxic apparatus was used to position the tip of the needle (26 gauge) at coordinates 0.4 mm anterior, 3.2 mm lateral, and 5.8 mm deep relative to bregma through a 1-mm craniotomy. ICH was produced by injection of 50 μL of fresh (non-heparinized) autologous whole blood into the right striatum at a constant rate of 8 μL/min. After a 10-min break, other 50 μL of blood were infused at the same rate for a total of 100 μL. The needle was left in place for 10 min after the infusion and was then withdrawn and the incision closed with sutures. Rats were randomly assigned to the sham or ICH group. Sham operation was restricted to needle insertion.

### Behavioral Testing

All behavioral testing were blinded to the group (ICH vs. sham) assignment and to neuro-imaging findings during the analysis.

#### Spontaneous Locomotion

Spontaneous locomotor activity was measured using an infrared actimeter (Panlab, Bioseb, Vitrolles, France). The apparatus consisted of a square arena (45 cm in length, 45 cm in width and 35 cm in height) with a black polymethyl methacrylate floor and transparent 34-cm-high polymethyl methacrylate walls. Rats were placed in the center of the arena and allowed to explore freely for 10 min. Activity was recorded by two rows of infrared photocell sensors and processed with Actitrack software (Bioseb). The total distance covered (in cm), the duration of inactivity (resting time, in seconds) and the number of rearings were collected.

#### Anxiety-Like Behavior

To check whether ICH did not cause alterations in anxiety like behavior, animals were tested using a modified version of the elevated plus maze (Pellow et al., 1985). The device consisted of two open arms (40 cm 20 cm) alternating at right angles with two arms enclosed by 40 cm high walls. The four arms delimited a central area of 20 cm^2^. The whole apparatus was placed 70 cm above the floor. Rats were placed in the center of the elevated plus maze, facing a closed arm, and were allowed to freely explore the maze for 10 min. The percentage of time spent in the open arms were measured.

#### Working Memory

The spontaneous alternation test was performed using a Y-maze (made of black wood) and a closed-circuit video camera (Ethovision XT, Noldus, Wageningen, The Netherlands). The three arms were of the same size (50*15*32 cm) and were oriented at an angle of 120° to each other. The Y-maze was placed in a room with no environmental cues. The rat was placed at the end of one arm and was allowed to move freely between the maze’s three arms for 8 min. A visit to an arm was scored when all four of the rat’s paws were within the arm area. The sequence of the arm visits was recorded, and an alternation response was scored when the animal entered the least recently visited arm. The alternation score was calculated as the ratio between actual alternations and possible alternations (defined as the total number of arm visits minus 2), multiplied by 100. This calculation was only performed for rats making more than fifteen arm visits (Hidaka et al., 2008).

#### Spatial Recognition Memory

Discrimination of novelty vs. familiarity can be studied by comparing exploring behavior in the three arms of Y-maze test. Each of the three arms was 35 cm long, 8 cm wide and 15 cm high. To differentiate the arms, three distal cues were placed on the walls around the Y-maze in the testing room and were kept in place throughout the behavioral testing period. Spatial memory was evaluated in a two-trial procedure with an inter-trial interval of 1 h. Three arms were designated: the starting arm, in which the rat started to explore (always open), the novel arm (which was closed in the first trial but open in the second trial) and the other arm (always open). The first trial (training session) lasted 10 min and enabled the rat to explore two arms of the maze (the start arm and the other arm). After a 1 h inter-trial interval, the second trial (test session) was performed; the rat was placed in the Y-maze in the same starting arm, with free access to all three arms for 5 min. Behavior was recorded with a video tracking system (Ethovision Noldus, The Netherlands) designed to automate testing in behavior experiments. The tendency to ignore the novel arm was determined by the percentage of time spent in the novel arm over those values in the two other arms during 2nd trial of the Y-maze test. A spontaneous tendency of animals to explore a novel environment as determined by the Y-maze test is generally regarded as a measure of spatial memory (Dellu et al., 1992).

#### Visual Recognition Memory

The novel object recognition test is based on the tendency of rats to explore a novel object rather than a familiar one. Rat were habituated to a square arena (50*50*25 cm) for 10 min on the first day. On day 2, rats were exposed to the arena containing two identical objects. On day 3, rats were exposed, in a first phase (“sample phase”), to two novel and identical objects for 15 min. After a 1 h resting time in their cage, they were placed back in the testing arena for 5 min (“test phase”), but a novel object had previously replaced one of the familiar objects. Performance of the rat was video recorded (Ethovision XT, Noldus, Wageningen, The Netherlands). Exploratory behavior was defined as the animal directing its nose toward the object at a distance below 2 cm. Any subject that failed to complete a minimum of 10 s of exploration during the sample phase was excluded from the analysis. The discernment index was calculated as the subtraction of the time spent exploring the novel object and the familiar object divided by the total time exploring either object (Antunes and Biala, 2012).

#### *In vivo* Magnetic Resonance Imaging

##### Image Acquisition

All rats underwent a 7-Tesla micro-MRI (7-Tesla; BioSpec 70/20, Bruker, Ettlingen, Germany) at days 0, 3, 7, and 3 months after ICH induction. Animals were anesthetized with isoflurane (1.5–2%) through spontaneous respiration and core temperature was maintained at 36–37°C throughout all surgical and MRI procedures. A cylindrical emitter antenna with a diameter of 72 mm and a cerebral receiving surface antenna both allow data recording. A birdcage radiofrequency coil and a surface coil were used for radiofrequency transmission and reception, respectively. Ear bars and a nose cone, through which the anesthetic gas mixture was supplied, were used to minimize head movement during MRI measurements. A 3-plane scout imaging sequence was obtained at the start of each MRI session to reproducibly position the animal in the magnet. For the current study, we used axial and coronal T2-weighted sequences [repetition time/echo time (TR/TE) = 5,000/77 ms, field of view (FOV) = 4*4 cm, matrix = 256*256, slice thickness = 1 mm, no gap, 20 slices].

##### Image Processing and Analysis: Initial Intracerebral Hemorrhage Volume and Cerebral Atrophy Assessment

We used MRI data obtained at days 0, 3, and 7 to quantify ICH volume. To do so, ICH was semi-automatically segmented using Mango© software. In Mango©, prior to any segmentation procedures, all T2-axial images underwent brightness/contrast enhancement using a contrast control tool to increase image quality and enhance background separation as much as possible. Thresholding was then applied, the values were automatically set (using the auto-threshold tool) to include only regions having pixels with high intensity levels, that isolated ICH, edema, and normal parenchyma. Region of interest (ROI) was then manually defined. Segmentations were independently analyzed by two experienced operators (L.P and F.A) with excellent interobserver agreement (*r* = 0.92). To ensure that all rats in the ICH group had a significant lesion, we excluded rats with initial ICH volume < 10 mm^3^.

We used indirect volume loss assessment with *in vivo* MRI as a marker of striatal and cerebral atrophy at 3 months and proceeded as follows. First, bias field inhomogeneity was corrected from T2-weighted images using N4 algorithm (Tustison et al., 2010). Then, images were denoised with a multiresolution non-local means filter (Coupé et al., 2012). Brain mask and label segmentation were automatically extracted with ANTS (Advanced Normalization Tools package) and from Cermep atlas (Lancelot et al., 2014). All MRI were visually checked and manually corrected when appropriate by two experienced operators (L.P and C.L). We first checked that there was no difference between Sham and ICH in the contralateral areas (see Supplementary Figure 1). The measurement was expressed as a ratio of the ispilateral volumes divided by the contralateral volumes for each ROI. To go further, we performed atrophy-related network analysis with Group-wise anatomical correlation matrix. Firstly, each ROI volume was normalized by dividing its volume by the whole brain volume. We tested whether there was significantly different anatomical correlation, represented by Fisher-transformed *Z*-values, between the Sham and ICH groups. Permutation tests were performed to statistically compare correlation matrices between the two groups. At first, PET images of each group were randomly permuted to make pseudo-random groups reassigned 10,000 times and from each paired group of rats to construct sampling distribution of correlation coefficients, interregional correlation matrices were calculated (Choi et al., 2015). Type I errors were determined by the comparison between the observed Z score for each connection and the Z score sampling distribution. A *p* < 0.05 was considered significant (Benjamini and Hochberg, 1995).

### Fluorodeoxyglucose—Positron Emission Tomography

Three months after surgery, cerebral metabolism was studied by performing a microPET scan (Inveon, Siemens) with an intravenous injection of fluorinated analog of glucose, the fluorodeoxyglucose ([18F] FDG) (34.76 ± 6.50 MBq; 300 μL in volume, IBA-CisBio, Saclay, France) in a sample of 12 representative animals. A 15-min PET scan was initiated 30 min after radiotracer injection. To assess changes in metabolism in the regions of interest (striatum, thalamus and hypothalamus, cortex and hippocampus), data from the scanner were formatted into three frames of 5 min. Those 3 frames were averaged and registered with SPM 12 (Welcome Department of Cognitive Neurology, London, United Kingdom)^1^. PET and CT images were rigidly co-registered with SPM 12. All CT scans were registered to an in-house CT template and brain masks were extracted and applied to PET images with ANTS (Advanced Normalization Tools package). PET images were registered with ANTS to their own T2-weighted image which were registered to Cermep rat atlas (Lancelot et al., 2014). FDG brain uptake values were expressed as Standardized Uptake Values (SUV) normalized to the average signal of the whole brain (SUVw). SUVw images were smoothed using an isotropic Gaussian filter [0.8 × 0.8 × 0.8 mm]. Voxel-based analysis with Statistical Parametric Mapping (SPM) was performed using SPM12 (Welcome Department of Imaging Neuroscience, London, United Kingdom) and the SAMIT toolbox (Vállez Garcia et al., 2015). For each voxel, two-sample *t*-tests were performed to compare ICH vs. sham animals. The *a priori* hypothesis was that the focal induction of ICH induces a diffuse reduction of cerebral metabolism (decreased cerebral [18F] FDG uptake) compared with the Sham group. The images generated by this post-treatment are statistical T-maps. They show the significant differences (expressed as *T*-values) between the two groups (ICH vs. sham) for each voxel registered onto a MRI rat brain template (Cermep). T-map data were thresholded at *p* < 0.05 (uncorrected) and an extent threshold of 200 voxels. Clusters with *p* < 0.05 corrected for family-wise error (FWE) were considered significant. To go further, brain metabolic connectivity was compared between Sham and ICH group. We tested whether there was significantly different connectivity, represented by Fisher-transformed *Z*-values, between the Sham and ICH groups. Permutation tests were performed to statistically compare correlation matrices between the two groups. At first, PET images of each group were randomly permuted to make pseudo-random groups reassigned 10,000 times and from each paired group of rats to construct sampling distribution of correlation coefficients, interregional correlation matrices were calculated (Choi et al., 2015). Type I errors were determined by the comparison between the observed Z score for each connection and the Z score sampling distribution. A *p* < 0.05 was considered significant (Benjamini and Hochberg, 1995).

### Histopathological Correlates

At the end of the protocol (i.e., 3 or 6 months for the sub-group with extended follow-up), rats were euthanized with an overdose of pentobarbital (200 mg/kg, intraperitoneal). For tissue staining procedure, rats were transcardially perfused with heparinized physiologic saline for 5 min and decapitated. Subsequently, brains were isolated and fixed in methacarn solution (60% methanol-30% chloroform- 10% acetic acid) at 4°C for 1 day, then in 70% ethanol at 4°C for 1–3 days, followed by paraffin embedding. Brains were serially sliced at 5 μm thickness. Sections of formalin-fixed paraffin embedded tissue were stained routinely with hematoxylin–eosin (H&E) for the identification of ICH and structural analysis of the tissue. Perls’ staining was done to screen for iron deposits using Tissue-Tek Prisma Plus automated Stainer (Sakura). Slides were placed in Potassium Ferricyanide solution (2% Potassium Ferricyanide –25 ml and 2% Hydrochloric acid –25 ml) for 20 min and then counterstained in Nuclear fast Red for 5 min, rinsed in distilled water, dehydrated and mounted.

### Statistical Analysis

Distribution of the data was established using the Kolmogorov-Smirnov test. All data are presented as mean ± SD or median [IQR] as appropriate. Statistical differences in data with normal distribution were analyzed with Student’s *t*-test, others with Mann-Whitney *U*-test. First, statistical analyses were performed on total population (ICH vs. sham) and then in sex-subgroup. All statistical tests were 2-sided and a *p*-value of < 0.05 was considered as statistically significant.

## Results

### Intracerebral Hemorrhage Characteristics and Locomotor Function at the Acute Phase

**FIGURE 2:**
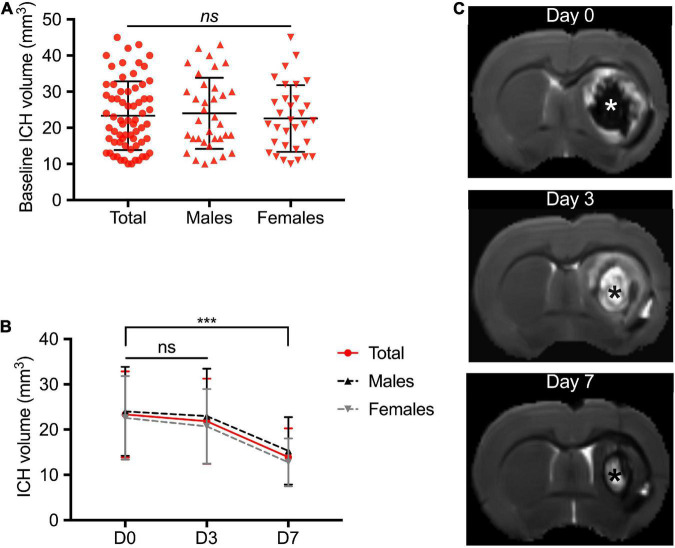
Temporal course of intracerebral hemorrhage volume in the acute phase. **(A)** Distribution of the ICH volume at day 0 in the total study population including male and female rats. Magnetic resonance imaging (MRI) was performed at 4 ± 1 h following ICH induction. **(B)** Temporal course of hematoma volume between day 0 and day 7. **(C)** Representative brain MRI axial T2 image of a female rat with right striatal ICH. Baseline ICH volume was 32 cm^3^. The asterisks showed the ICH core. ICH was defined at D0 by a T2 hyposignal area surrounded by an hypersignal rim that corresponds to peri-hematomal edema. White signal area observed at D3 and D7 within the core of ICH is believed to be due to presence of extracellular methemoglobin following erythrolysis within the hematoma core (Knight et al., 2008; Dang et al., 2017). Symbols and bars correspond to the mean value and the SD. The *p*-value came from a Student’s *t*-test (***p < 0.001, ns, not significant).

Among the 114 ICH animals, 3 died before the first MRI and 7 had a baseline ICH volume < 10 mm^3^. Therefore, 104 animals (53 males, 64 ICH) were included in the study. In the ICH group, initial mean hematoma volume was 23.35 ± 9.50 mm^3^ (Figure 1A). We did not find significant differences between males and females (24.03 ± 9.82 mm^3^ vs. 22.60 ± 9.23 mm^3^, respectively, *p* = 0.43) (Figure 2A). The ICH volumes were similar at day 0 and day 3 and decreased significantly at day 7 (13.91 ± 0.9 mm^3^, *p* < 0.0001 with day 0 as reference). The same temporal course was found for both males and females (Figures 2B,C).

**FIGURE 3:**
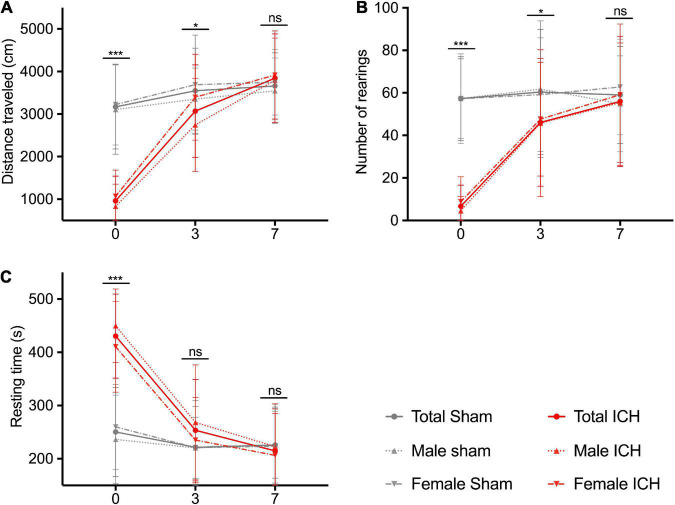
Spontaneous locomotion recovery in the acute phase. Traveled distance in cm **(A)**, number of rearings **(B)**, and resting time in seconds **(C)**. Red and gray lines correspond to the ICH groups and the sham groups performances, respectively. Solid, dotted and hashed lines correspond to total population, male and female rats, respectively. Measurements were assessed at days 0, 3, and 7. Symbols and bars correspond to the mean value and the SD. The *p*-value came from a Student’s *t*-test (***p ≤ 0.001, *p ≤ 0.05, ns, not significant).

Figure 3 shows the spontaneous locomotion of animals (total population and sex-subgroups) between days 0 and 7. At day 0, ICH rats showed severe locomotor impairment with a significant decrease in the traveled distance (ICH group vs. sham group, *p*-value: 961 ± 69 vs. 3,168 ± 156 cm, *p* < 0.0001), a lower number of rearings (6.7 ± 1.2 vs. 57.3 ± 3.1, *p* < 0.0001) and a longer resting time (430 ± 9.3 vs. 250 ± 13 s, *p* < 0.0001). At day 3, ICH rats showed a partial recovery but were still significantly impaired. We observed a lower traveled distance (ICH group vs. sham group, *p*-value: 3,069 ± 131 vs. 3,548 ± 158 cm, *p* = 0.024), a lower number of rearings (46.1 ± 3.6 vs. 60.9 ± 4.7, *p* = 0.016) and a still longer resting time: 253 ± 11 vs. 222 ± 12 s, *p* = 0.07). At day 7, ICH rats showed complete recovery of locomotor function (traveled distance: 3,662 ± 123 vs. 3,844 ± 121 cm, *p* = 0.42/number of rearings: 56 ± 3.6 vs. 59 ± 3.6, *p* = 0.58/resting time: 215 ± 9 vs. 226 ± 11 s, *p* = 0.46). In sex sub-group analysis, males and females were equally injured at day 0. The females tended to recover faster than the males, with spontaneous locomotor performances comparable to their sham group from day 3 (traveled distance and resting time, *p* < 0.05).

### Intracerebral Hemorrhage Induces Long-Term Cognitive Impairment

**FIGURE 4:**
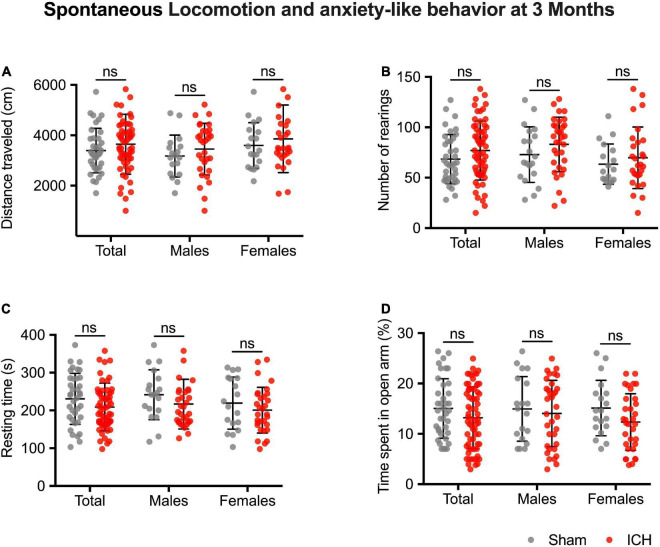
Long-term spontaneous locomotion and anxiety-like behavior. Traveled distance in cm **(A)**, number of rearings **(B)**, and resting time in seconds **(C)**. Anxiety-like behavior **(D)**, values correspond to the time spent in the novel arm, expressed in %. Gray and red symbols correspond to sham and ICH rats, respectively. Black bars correspond to the mean value and the SD. The *p*-value came from a Student’s *t*-test (ns, non-significant).

None of the included animals died between ICH induction and the follow-up assessments. At three months, ICH rats had similar spontaneous locomotor performances compared to the Sham rats (ICH group vs. sham group, difference between means, *p*-value; distance traveled: 3,646 ± 155 vs. 3,393 ± 146 cm, 253 ± 228, *p* = 0.27/number of rearings: 76.9 ± 3.7 vs. 68.4 ± 3.9, 8.5 ± 5.7, *p* = 0.13/resting time: 209 ± 8 vs. 230 ± 11 s, –22 ± 13, *p* = 0.10) (Figures 4A–C). They also did not exhibit more anxiety-like behavior (% of the time spent in open arm: 13.2 ± 0.8 vs. 15.0 ± 1%, –1.8 ± 1.2, *p* = 0.14) (Figure 4D). Results were similar in both males and females in sex-subgroup analysis.

**FIGURE 5:**
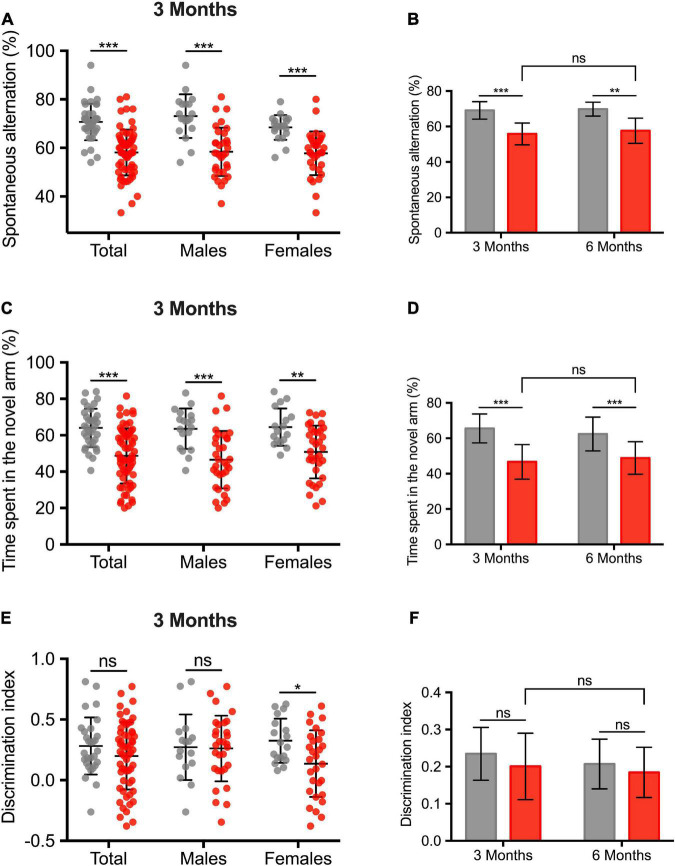
Long-term cognitive impairment after intracerebral hemorrhage induction. **(A)** Assessment of the working memory, expressed in% of spontaneous alternation. **(C)** Assessment of the spatial and hippocampal memory. Values correspond to the time spent in the novel arm, expressed in%. **(E)** Assessment of the visual recognition memory. **(B,D,F)** Comparison of cognitive performances between 3 and 6 months in a subgroup analysis of 24 animals (10 sham rats, sex ratio 1:1). Gray and red barplots correspond to sham and ICH rats, respectively. Black bars correspond to the mean value and the SD. The *p*-value came from a Student’s *t*-test (***p ≤ 0.001, **p ≤ 0.01, *p ≤ 0.05), ns: not significant).

Figures 5A,C,E shows the cognitive impairment at 3 months (total population and sex-subgroups). The ICH group exhibited a cognitive impairment in several domains. An alteration of the working memory was observed (% of spontaneous alternation: 58.1 ± 1.2 vs. 70.7 ± 1.2%, –12.6 ± 1.8, *p* < 0.001). Spatial recognition memory was also altered (% of time spent in the novel arm: 48.7 ± 1.9 vs. 64 ± 1.8%, –15.3 ± 2.9, *p* < 0.001). We did not find any sex effect regarding the working and spatial recognition memory. Regarding visual recognition memory, ICH female rats showed a deficit in visual recognition memory (discrimination index: 0.14 ± 0.05 vs. 0.33 ± 0.04, –0.19 ± 0.07, *p* = 0.013). In the subgroup of 24 animals followed up to 6 months, spontaneous alternation test and the Y-maze spatial test were still impaired compared to the 3-months assessment (Figures 5B,D,F).

### Intracerebral Hemorrhage Induces Long-Term Structural and Metabolic Alterations

**FIGURE 6:**
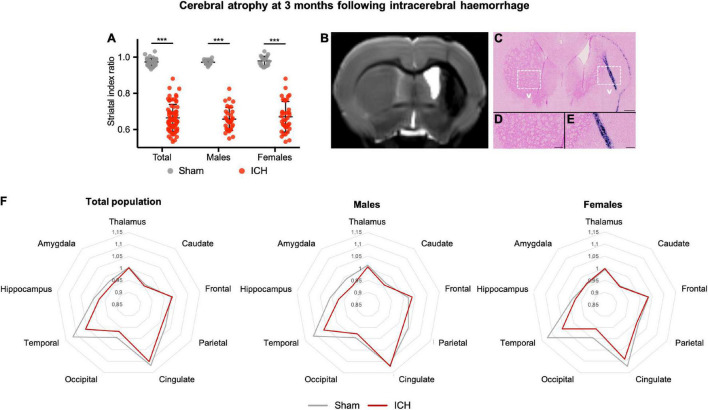
Long-term cerebral atrophy and histological correlates after intracerebral hemorrhage. **(A)** Striatal index ratio defined as the volume of the ipsilateral striatum/volume of the contralateral striatum. Gray and red symbols correspond to sham and ICH rats, respectively. Black bars correspond to the mean value and the SD. The *p*-value came from a Student’s *t*-test (***p ≤ 0.001). **(B)** Representative MRI axial T2 image of the same female rat, as described in Figure 1. One can notice the ICH sequel in the right striatum with an atrophy and hydrocephalus. **(C–E)** Perl’s staining showing iron accumulation within the ICH scar but also within the corpus callosum. One can notice the loss of the alveolar structure of the injuried striatum compared to the contralateral one. Scale bars = 500 μm for **(C)** and 100 μm for **(D,E)**. ICH, intracerebral hemorrhage. **(F)** Radar chart of the different brain structures from the Cermep atlas in male and female rats. For each structure, the results are expressed as the ratio of ipsilateral/contralateral. Gray and red lines correspond to sham and ICH rats, respectively.

The ICH group showed a severe ipsilateral atrophy as compared to the sham group (mean index ratio 0.66 ± 0.05 and 0.97 ± 0.02 for ICH and sham group, respectively, *p* < 0.001) (Figures 6A,B). Striatal atrophy was observed in both males and females without sex effect (*p* < 0.001). Histological examination of ICH rats confirmed the severe atrophy of the ipsilateral striatum characterized by a loss of alveolar architecture of the tissue. Perl’s staining showed the presence of numerous iron deposits in and around the ICH site (Figures 6C–E). Brain labels extracted from Cermep atlas showed that atrophy was not restricted to the striatum (Figure 6F). Limbic system structures (hippocampus and amygdala) and cortical areas (temporal, occipital and parietal) were atrophied. In males, atrophy was observed in areas involved in the limbic system ipsilateral amygdala (mean index ratio 0.94 ± 0.02 and 1.00 ± 0.04 for ICH and sham group, respectively), hippocampus (mean index ratio 0.96 ± 0.03 and 1.01 ± 0.03 for ICH and sham group, respectively) and temporal lobe (mean index ratio 1.05 ± 0.02 and 1.12 ± 0.02 for ICH and sham group, respectively), all *p*-value < 0.05 compared to sham animals (a trend was observed in the frontal lobe: *p* = 0.07). In females, atrophy was observed in temporal (mean index ratio 1.05 ± 0.04 and 1.13 ± 0.04 for ICH and sham group, respectively), occipital (mean index ratio 0.95 ± 0.03 and 1.00 ± 0.02 for ICH and sham group, respectively) and cingulate lobes (mean index ratio 1.08 ± 0.03 and 1.13 ± 0.04 for ICH and sham group, respectively), all *p*-value < 0.05 compared to shams. Atrophy-related network analysis showed significantly increased anatomical correlation in ICH group compared with Sham group were found between right parietal cortex and right cingulate cortex and between right parietal cortex and left thalamus. Significantly reduced anatomical correlation in ICH group compared with the sham group were found between 23 ROI pairs. So, anatomical correlation between right striatum and 10 ROI (left striatum, right occipital, left and right for cingulate cortex, for temporal cortex, for occipital cortex and for amygdala) were significantly reduced in ICH group compared to the Sham group. Anatomical correlation were also reduced between right cingulate cortex and right hippocampus, right frontal cortex and right hippocampus, right frontal cortex and right amygdala, temporal cortex left and temporal cortex right, right temporal cortex and right parietal cortex, right temporal cortex and right thalamus, left cingulate cortex and left temporal cortex, left cingulate cortex and right hippocampus, left cingulate cortex and left hippocampus, right thalamus and right hippocampus, right thalamus and left hippocampus, right thalamus and right amygdala and left thalamus and right hippocampus (see Supplementary Figure 2).

**FIGURE 7:**
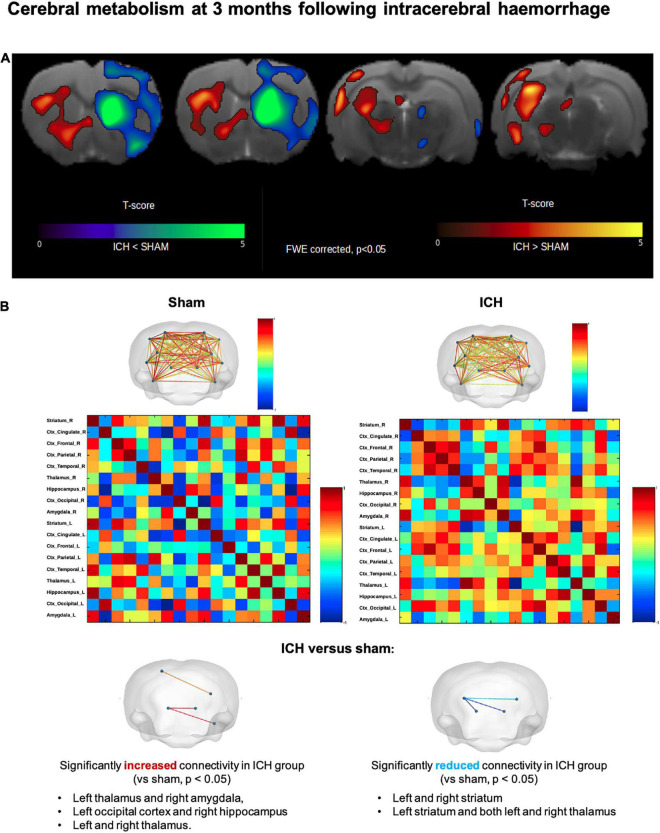
Long-term cerebral hypometabolism and network connectivity changes after intracerebral hemorrhage. **(A)** Results of 18-FDG PET voxel-based analysis, overlaid onto Cermep MRI template, comparing ICH and sham rats (males and females). Decrease in metabolism (*p* < 0.05 FWE-corrected) in ICH rats compared to sham rats was found in right striatum, right thalamus, right hypothalamus, right parietal, frontal and temporal lobes. Increase in metabolism (*p* < 0.05, FWE-corrected) in ICH rats compared to sham rats was found in contralateral region (left striatum, left thalamus, left temporal lobe, left parietal lobe, left occipital lobe, left amygdala, and left hippocampus). In the contralateral hemisphere (left), we observed a paradoxal hypermetabolism involving the following structures: caudate putamen, thalamus, temporal lobe, parietal lobe, amygdala and hippocampus. **(B)** Metabolic activities of 18 brain regions based on Cermep atlas were correlated to each other for ICH and sham groups. Significantly increased connectivity (*p* < 0.05) in ICH rats was found between left thalamus and right amygdala, left occipital lobe and right hippocampus and between left thalamus and right thalamus. Significantly decreased connectivity (*p* < 0.05) in ICH rats was found between left striatum and right striatum, left striatum and right thalamus and between left striatum and left thalamus. ICH, intracerebral hemorrhage.

Regarding the brain metabolic assessment (Figure 7), ICH (males and females) group exhibited a severe decrease in glucose uptake at the ipsilateral striatum compared to the sham group. In the ICH group, the voxel-based analysis also showed that the hypometabolism was not restricted to striatum. Hypometabolism was also observed in adjacent deep basal ganglia (ipsilateral thalamus, hypothalamus, caudate and putamen) as well as ipsilateral parietal, frontal and temporal lobes. In the contralateral hemisphere (left), we observed a hypermetabolism involving the following structures: caudate putamen, thalamus, temporal lobe, parietal lobe, amygdala and hippocampus (all *p* < 0.05 FWE-corrected, T-map details are reported in Figure 7A). We additionally provide mean SUV for each group (with Sham vs. ICH comparison) in the Supplementary Table 1. In sex subgroup analysis, male ICH rats exhibited a significantly decrease in glucose uptake in the hippocampus whilst ICH females exhibited a hypometabolism in the ispilateral amygdala. Brain metabolic connectivity analysis showed significantly increased signal in the ICH group compared with sham group, between the following areas: left thalamus and right amygdala, left occipital cortex and right hippocampus, and between left and right thalamus. Besides, significantly reduced metabolic connectivity in the ICH group compared with the sham group were found between: left and right striatum, left striatum and between left striatum and left thalamus (Figure 7B).

## Discussion

Several results emerged from this a large cohort of healthy male and female ICH rats: (i) We confirmed that ICH induces long-term multidomain CI even in healthy young rats; (ii) a focal striatal lesion induces a long-term remote alteration of the brain structure/function network involving the limbic system and cortical areas, and (iii) behavioral and radiological trajectories were similar between males and females despite some sex specificities.

Our results emphasize the presence of long-term CI after ICH, including both hippocampal and non-hippocampal aspects of cognition. This point contrasted with both spontaneous early locomotor recovery and significant spontaneous blood resorption (˜40% of the initial lesion) during the short-term period (from D0 to D7). Cognitive assessment was not confounded by motor impairment, nor anxiety. In sub-group analyses, we did not observe worsening of cognition between 3 and 6 months after ICH induction. This suggests that, in our model and experimental conditions, ICH itself was responsible for CI, rather than triggering a progressive decline. We aimed to study the specific effect of ICH on cognitive functions. Therefore, we used healthy rats in the young adult stage, devoid of underlying brain parenchymal or small vessel diseases. Future works are needed to determine to what extent the inclusion of disease-relevant comorbidities and risk factors, such as hypertension and aging, can modify the cognitive trajectory after ICH. Even if ICH-related CI is well documented in humans, to our knowledge, only two studies have assessed the cognitive functions after ICH in rats (Hartman et al., 2009; MacLellan et al., 2009). In the first one, cognitive tests on learning and memory, including spontaneous alternation, elevated plus maze, open-field, Morris water maze, T-maze and the radial arm maze, were cross sectionally conducted 1–7 months after ICH (MacLellan et al., 2009). In a cohort of 12 rats, no significant learning or memory deficits were observed after ICH. In the second study, investigators found significant learning deficits in a cohort of 17 rats at 2 weeks but not at 8 weeks (Hartman et al., 2009). These results contrast with ours but some limitations hamper to draw solid conclusion, including the inclusion of males only, the small effective size (*n* = 12 and 17, respectively), the small size of ICH lesions (18.24 ± 1.92 mm^3^ and 4.02 ± 2.1 mm^3^) and heterogeneity in outcome assessment methods. This highlights the need to develop comprehensive and standardized method to assess cognition in experimental stroke models (Hietamies et al., 2018).

Our data from brain atrophy and metabolism measurements contribute to deepen our understanding of the mechanisms involved in ICH-related CI: we found a remote alteration of both brain structure and function that went beyond the blood injection site. Focal ICH in the striatum induced a loss of one third of the structure volume, accompanied with severe hypometabolism. Histological examinations showed a loss of volume and architectural integrity of the striatum associated with an abundant sequestration of iron. One can hypothesize a direct involvement of iron neurotoxicity in CI (Salvador et al., 2010). More strikingly, we found that structural and functional consequences of ICH spread beyond the striatum following a network organization: thalamus, the limbic system (amygdala and hippocampus) and cortical areas (especially the temporal, occipital and parietal lobes). These radiological findings are consistent with our behavioral assessments (impairment of working, spatial, hippocampal and, in a lesser extent, visual memory) but also with previous literature on striatum function and its interaction with other cerebral areas (Rolls, 1994; Rektor et al., 2004; Groenewegen and Trimble, 2007; Provost et al., 2015). Taken together, our data confirm that the role of striatum is not limited to motor functions but also plays an integrative role in cognitive processes through extensive connections with key structures involved in the cognitive process.

To support these findings, we performed brain metabolic connectivity analysis and atrophy-related network analysis. We provide a description of the impaired or increased network connectivity in ICH rats that are consistent with current knowledge in brain connectomics disorders (Fornito et al., 2015). We observed a structural and functional deterioration of areas (especially limbic system structures) connected to the affected site, but remotely from the initial insult. This suggests the occurrence of maladaptive process such as diaschisis and/or transneuronal degeneration. Given the cross-sectional design of our structural and functional brain investigation at 3 months, the respective contribution of these two processes is unclear. Further longitudinal analysis will be required. On the other side, we observed an increase in activity and functional connectivity in the contralateral hemisphere (especially involving the limbic system; thalamus, amygdala, hippocampus and temporal lobe) suggestive of a neural compensation process. In regard to our behavioral investigation, we hypothesize that this compensation improved spontaneous locomotion, but not the recovery of more complex functions, such as cognition. Further studies are required to better investigated the histopathological correlates of observed remote degeneration and adaptative process, including neuronal shrinkage; reductions in dendrite and synapse number; alterations of axonal myelin content and fiber number; and neuronal death.

The design of our study allowed us to study the sex effect. The recent literature on ICH and more generally experimental models reports a sex bias given the under-representation of female animals (Zucker and Beery, 2010; Sugimoto et al., 2019). Even if male and female rats share common outcome trajectories, we observed some differences. First, female rats recovered faster than male rats regarding spontaneous locomotion within the first week following ICH induction. Second, only female rats had alteration of the visual recognition memories. Third, male and female rats share distinct pattern of structural and functional alterations. Indeed, beside the striatum, atrophy and hypometabolism were mostly found in limbic system structures in males and in cortical area in females. To what extent these differences could interfere with the effect of pharmacomodulation is unknown but should be taken into account in preclinical and clinical studies.

Our findings also raise concern regarding assessment of treatments efficacy in ICH models. Indeed, most of stroke model focused on motor functions in the first month following brain injury in rodents (Hietamies et al., 2018). Given that animals showed an early spontaneous motor recovery, the assessment of a potential drug effect solely based on motor function is questionable and CI assessment should be required.

Our study has limitations. Despite a large panel of behavioral assessments, fine motor alterations (e.g., motor coordination, balance and muscle strength) may have been missed. We did not investigate some cognitive domains such as visuospatial memory, cognitive flexibility or learning.

Our study has also strengths. We used a large sample size to ensure a reliable detection of long-term cognitive deficits. The volumes of the ICHs were large enough to mimic bleeding in humans. To minimize measurement bias and subjectivity of scales, we used fully automated method to assess spontaneous locomotion and cognition. We used semi-automated method for volume segmentation and to obtain brain mask atlas. We combined structural and functional data with MRI and 18FDG-TEP to further modelize the structure-function relationship of post-ICH cognitive performance. Finally, to assess sex effect, we included both males and females, so our design was consistent with the “Sex and Gender Equity in Research” guidelines (Heidari et al., 2016).

## Conclusion

In a young and disease-free rat brain, we showed that a focal striatal ICH provokes long term multidomain CI and remote cerebral atrophy and hypometabolism, especially involving limbic system and cortical areas. These radiological findings deepen our understanding of ICH-related CI and may serve as biomarkers in the view of future therapeutic intervention.

## Data Availability Statement

The raw data supporting the conclusions of this article will be made available by the authors, without undue reservation.

## Ethics Statement

All experiments were approved by the national Ethical Committee in Animal Experimentation (CEEA, Comité d’Ethique en Experimentation Animale), from the French Ministry for Education and Research (agreement number: APAFIS#14066-2018031312529642v3).

## Author Contributions

LP, VB, and CC contributed to the conception and design of the study. LP, CL, and FA contributed to the acquisition and analysis of the data. LP, CL, VB, and CC drafted the text and prepared the figures. RB provided a review of the manuscript. All authors contributed to the article and approved the submitted version.

## Conflict of Interest

The authors declare that the research was conducted in the absence of any commercial or financial relationships that could be construed as a potential conflict of interest.

## Publisher’s Note

All claims expressed in this article are solely those of the authors and do not necessarily represent those of their affiliated organizations, or those of the publisher, the editors and the reviewers. Any product that may be evaluated in this article, or claim that may be made by its manufacturer, is not guaranteed or endorsed by the publisher.

## Footnotes

1
http://www.fil.ion.ucl.ac.uk/spm/
