# Supplementary material for: Intracerebral Hemorrhage-Induced Cognitive Impairment in Rats Is Associated With Brain Atrophy, Hypometabolism, and Network Dysconnectivity
Source: Front Neurosci. 2022 Jun 30;16:882996. doi: 10.3389/fnins.2022.882996 (PMC9280302; doi:10.3389/fnins.2022.882996)
Supplement: Supplementary file 1 [file Data_Sheet_1.PDF]

Supplementary Material

Supplementary Figure 1: Comparison of contralateral brain areas volumes between Sham and ICH group.

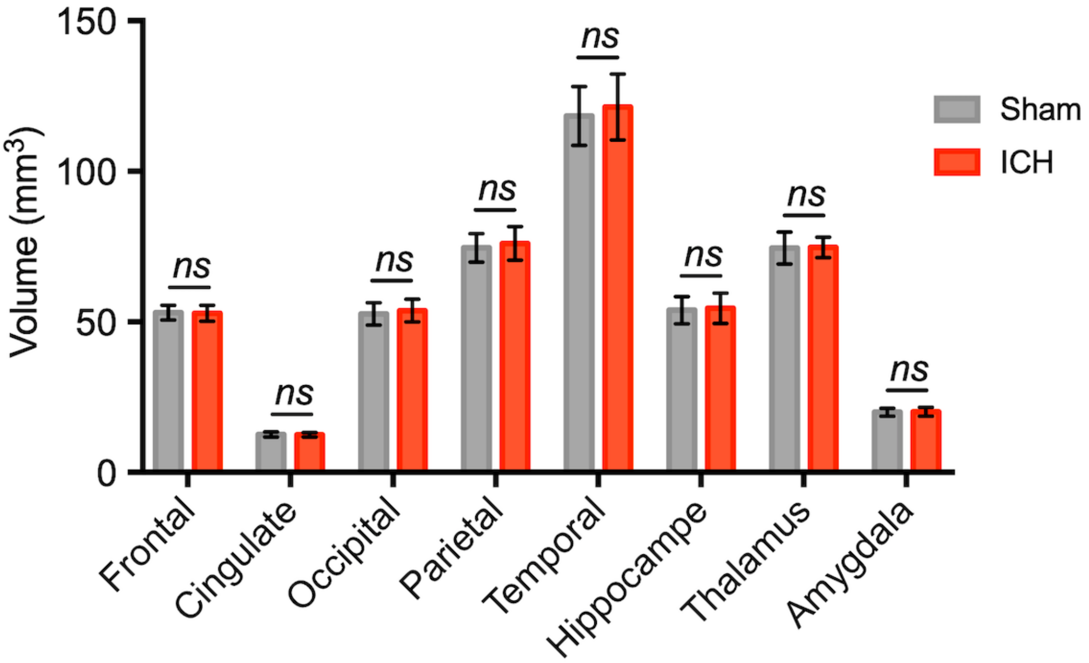

Supplementary Figure 2: Atrophy-related network analysis at 3 months after ICH induction.

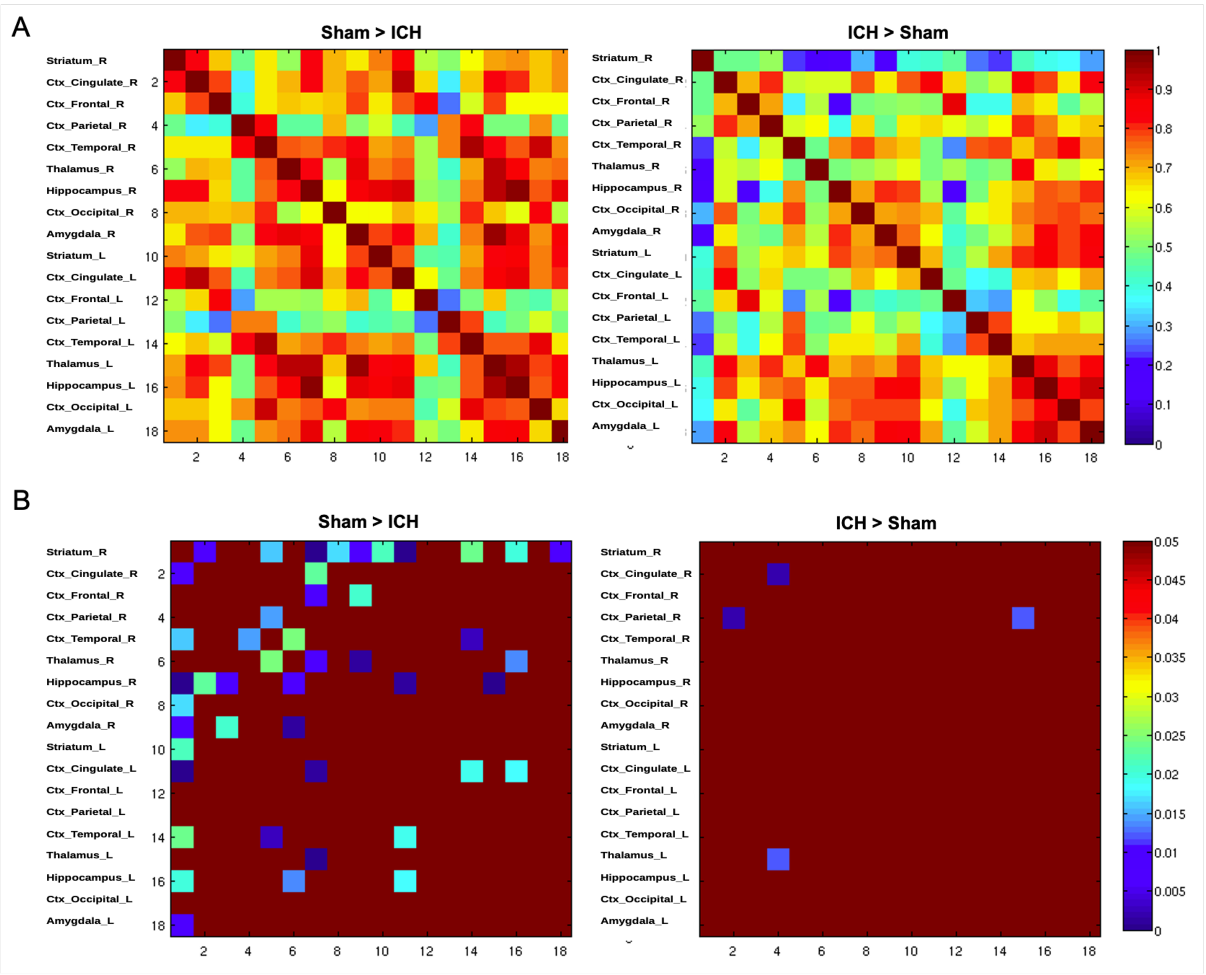

Legend: Atrophy-related network analysis was performed on volume of 18 brain regions based on Cermep atlas. First, the volumes of the regions were normalized by whole brain volume. Pairs of volumes were correlated for ICH and sham groups. We performed a permutation analysis (10,000 permutations) to statistically compared correlation matrices between ICH and sham group. A p<0,05 was considered significant difference. Panel A provides correlation values and Panel B provides *p* values.

Supplementary Table 1: Mean Standardized Uptake Values per group (Sham versus ICH).

SUVw	Caudate putamen right	cCngulate cortex right	Frontal cortex right	Parietal cortex right	Temporal cortex right	Occipital cortex right	Thalamus right	Hippocampus right	Amygdala right
Sham	1,15	1,33	1,11	1,16	1,07	1,11	1,09	0,94	0,88
ICH	0,96	1,31	1,06	1,11	1,03	1,09	1,06	0,96	0,88
p-value	** 0,004	ns 0,60	* 0,03	ns 0,05	* 0,02	ns 0,29	ns 0,12	ns 0,48	ns 0,97

SUVw	Caudate putamen left	Cingulate cortex left	Frontal cortex left	Parietal cortex left	Temporal cortex left	Occipital cortex left	Thalamus left	Hippocampus left	Amygdala left
Sham	1,14	1,31	1,14	1,18	1,10	1,12	1,09	0,93	0,87
ICH	1,23	1,32	1,15	1,21	1,12	1,15	1,13	0,10	0,91
p-value	* 0,01	ns 0,64	ns 0,81	ns 0,07	ns 0,31	ns 0,12	** 0,002	** 0,006	ns 0,06

Legend: For each rat, SUV (Standardized Uptake Values) were normalized to the average signal of the whole brain (SUVw). Mean SUVw were computed for sham and ich group. Approximative two-sample Fisher-Pitman Permutation test with resample = 10,000 was performed on SUVw data between Sham and ICH group.
